# Emulsion Electrospinning of PLLA/PVA/Chitosan with *Hypericum perforatum* L. as an Antibacterial Nanofibrous Wound Dressing

**DOI:** 10.3390/gels9050353

**Published:** 2023-04-22

**Authors:** Cláudia Mouro, Ana P. Gomes, Isabel C. Gouveia

**Affiliations:** FibEnTech—Fiber materials and Environmental Technologies Research Unit, Faculty of Engineering, University of Beira Interior, 6200-001 Covilhã, Portugal; d1684@ubi.pt (C.M.); anapaula@ubi.pt (A.P.G.)

**Keywords:** emulsion electrospinning, gel-like suspensions, crude plant extracts, *Hypericum perforatum* L., antimicrobial wound dressing, chronic wounds

## Abstract

Chronic wounds are one of the most severe health problems that affect millions of people worldwide. These types of injuries impair healing and lead to life-threatening complications. Therefore, suitable wound dressing materials are essential to prevent the risk of infection and to provide an excellent healing environment. The present research reports the development of an electrospun Poly (L-lactic acid) (PLLA)/Poly (vinyl alcohol) (PVA)/Chitosan (CS) wound dressing material, produced via emulsion electrospinning in a single step using homogeneous gel-like suspensions of two different and incompatible polymer solutions. The electrospun PLLA/PVA/CS fiber mats were loaded with two different amounts of *Hypericum perforatum* L. (HP) (2.5% and 5.0% owf). The results revealed that the produced electrospun PLLA/PVA/CS fiber mats displayed ideal properties as a wound dressing due to a total porosity, wettability, water vapor transmission rate (WVTR), and swelling properties similar to those reported for the extracellular matrix (ECM) of the skin, mainly when 2.5% owf HP was incorporated. Moreover, the electrospun PLLA/PVA/CS fiber mats containing HP were able to prevent the growth of gram-positive bacterium *Staphylococcus aureus* (*S. aureus*) without causing cytotoxicity to normal human dermal fibroblasts (NHDF). These findings suggest that these electrospun dressing mats are helpful for preventing wound infections as well as an appropriate support and microenvironment for wound healing.

## 1. Introduction

Skin damage usually results in a dynamic and complex process mediated through a coordinated cascade of biological events, which are traditionally divided into four distinct but overlapping phases: hemostasis, inflammation, proliferation, and maturation [1,2,3,4,5]. Nevertheless, the healing process can be severely impaired by several factors, and consequently, the wound repair cannot proceed in a timely and orderly manner, resulting in chronic, hard-healing wounds [3,4,6].

Chronic wounds have become a severe public healthcare concern and are strongly associated with significant morbidity and mortality [1,2,3,4,6,7]. Nowadays, more than 10 million people around the world are affected by this problem, and it is expected to increase due to an aging population and the sharp rise of diabetes and obesity [4,7,8]. Their higher incidence is generally associated with venous or arterial insufficiency, prolonged pressure, or diabetes [7,9,10].

So far, diverse approaches have been employed for the management of these wounds. Among them, the application of different types of antimicrobial dressings (e.g., films, sponges, hydrogels, hydrocolloids, and membranes), which helps in reducing inflammation and preventing wound contamination, has been regarded as a useful practice to treat and avoid chronic wounds occurring [5,8,11]. However, successful healing of chronic wounds often requires long periods of treatment, and as expected, their management is costly. To overcome these limitations, suitable nanofibrous wound dressing membranes that actively promote wound regeneration have attracted researchers’ attention due to their therapeutic potential. More specifically, wound dressings which protect the wound from external threats and subsequent infection, while absorbing wound exudate, creating and maintaining a moist wound environment, as well as nutrient and gas exchange have been developed to encourage the healing process [5,12,13,14].

Nowadays, many different techniques are being employed to produce micro to nanoscale materials, including electrospinning, phase separation, and self-assembly. Nevertheless, electrospinning has become one of the most desirable and attractive methods to develop nanofiber-based dressings, due to its remarkable properties, such as the extremely high surface area to volume ratio and high porosity, as well as the potential of mimicking the skin extracellular matrix (ECM) composition. These features are essential to keep an appropriate microenvironment for cell adhesion and proliferation [4,5,8,12,13,14,15,16]. Moreover, electrospinning allows the incorporation of bioactive agents into the electrospun nanofibers to enhance the functional and biologic performance of these wound dressing materials [4,5,8,12]. Numerous bioactive compounds, including antibiotics, antimicrobials, anti-inflammatories, analgesics, vitamins, growth factors, and natural agents such as medicinal plants, have been considered as promising supplements for the management and treatment of wounds [3,5]. Furthermore, researchers have recently identified the unique ability of electrospinning to incorporate sensors into membranes for detecting human motions as well as monitoring sports performance and health [17,18,19]. In fact, the incorporation of sensors into the electrospun membranes has emerged as a key approach for monitoring the wound microenvironment and status, as well as regulating the sustained release of bioactive compounds, providing ideal healing conditions [5].

**Figure 1:**
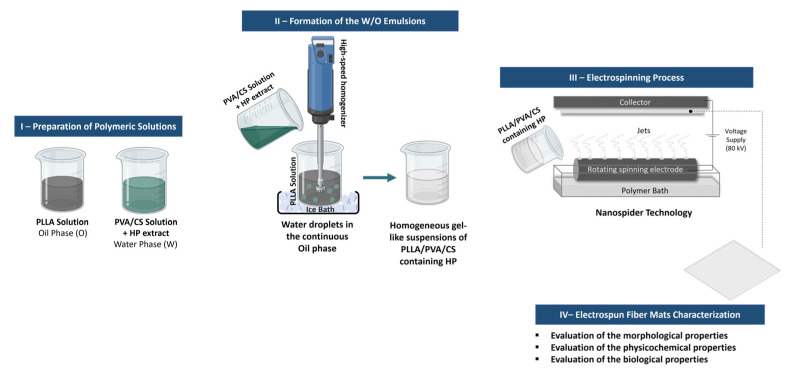
Schematic representation of the present study: production of the electrospun Poly (L-lactic acid) (PLLA)/Poly (vinyl alcohol) (PVA)/Chitosan (CS) fiber mats containing *Hypericum perforatum* L. (HP) via emulsion electrospinning using homogeneous gel-like suspensions.

Accordingly, this research work describes the innovative use of the Nanospider technology, a modified electrospinning technique based on a rotating spinning electrode immersed into a container with the polymeric solution to produce electrospun Poly (L-lactic acid) (PLLA)/Poly (vinyl alcohol) (PVA)/Chitosan (CS) fiber mats containing *Hypericum perforatum* L. (HP), St. John’s Wort, via emulsion electrospinning using homogeneous gel-like suspensions of PLLA, PVA, and CS polymer solutions, shown in Figure 1 [3,20]. In addition, the Nanospider makes it possible to produce multiple Taylor cones at the same time; hence, this technique is highly productive, although it requires the use of a higher voltage.

HP has been applied to the treatment of different skin disorders such as eczema, burns, and diabetic wounds [3,20]. Moreover, different bioactive metabolites such as naphthodianthrones, phloroglucinols, flavonoids, bioflavonoids, and phenylpropanoids have been found in HP extracts and associated with antifungal, antimicrobial, antiviral, and anti-inflammatory activities [3]. In turn, PLLA, a synthetic tissue-compatible polymer approved by the US Food and Drug Administration (FDA) for direct contact with biological fluids and widely used for biomedical field applications, was used to provide mechanical protection against external threats and prevent fluid loss from the wound surface due to its inherent hydrophobicity, high biocompatibility and biodegradability, as well as outstanding mechanical properties and strength [21,22]. Additionally, CS was blended with a carrier polymer such as the highly hydrophilic, non-toxic, biocompatible, and biodegradable PVA to suppress the difficulty of producing pure CS nanofibers by electrospinning due to its ability to form a viscous gel in an acidic aqueous solution. PVA presents an exceptional ability to act as an emulsifying and dispersing agent promoting the formation of stable emulsions, while CS has revealed promising wound-healing abilities and scar-prevention properties because it can stimulate collagen synthesis and displays antimicrobial and hemostatic properties [23,24,25,26,27,28]. In addition, CS is a natural, biodegradable, biocompatible, abundant, and versatile biopolymer, and due to its unique properties, diverse CS-based wound dressings have been produced in several forms, such as films, sponges, hydrogels, and nanofibrous membranes [23,24,25,26,27,28]. Herein, the electrospun PLLA/PVA/CS materials prepared via emulsion electrospinning using homogeneous gel-like suspensions were loaded with crude HP extracts to prevent bacterial growth and facilitate the healing process. The effective loading of crude HP extracts into PLLA/PVA/CS gel-like suspensions was evaluated, and the results revealed that this cost-effective and eco-friendly natural bioactive agent was successfully applied to these wound-dressing materials. Furthermore, emulsion electrospinning was revealed to be an alternative approach to produce, in a single step, wound dressings made from gel suspensions using multiple polymers, displaying antimicrobial activity. In addition, the emulsion electrospinning does not require a specific needle set-up; hence, this methodology becomes a viable option to circumvent some of the limitations presented by the electrospinning techniques that use two or more syringes to produce nanofibers from immiscible polymers, such as PLLA, PVA, and CS [29].

## 2. Results and Discussion

### 2.1. Quantification of Total Hypericin (Hyp) Content in Crude Ethanol Extract of HP

The content of Hyp present in the ethanol extract of HP was measured using the spectrophotometric method. Hyp is one of the main bioactive agents present in HP extracts, and it is responsible for several therapeutic properties. As seen in Table 1, the total Hyp content was 0.30 ± 0.07 (%), according to the calibration equation (y = 0.0002x − 0.0234) with an r^2^ = 0.99. These results showed a linear relationship between the concentration of Hyp and absorbance at 587 nm. Similar results have been already reported by Pourhojat et al., who obtained a total Hyp content from HP extract of 0.23 ± 0.06 (%) [30]. Therefore, the extraction method was efficiently accomplished.

### 2.2. Determination of Minimum Inhibitory Concentration (MIC) of Crude HP Extract

The MIC value of crude ethanol extract of HP against *S. aureus* (gram-positive bacterium) was found to be 2.50 mg/mL. According to Okmen et al., methanol HP flower extracts gave a MIC value of 3.25 mg/mL against *S. aureus*—17 and a MIC value of 1.63 mg/mL against *S. aureus*—18 [31]. On the other hand, Reichling et al. obtained MIC values of 2.50 mg/mL and in the range of 1.3–2.50 mg/mL, for several filtered teas and loose teas from HP against *S. aureus*, which were similar to the MIC value obtained in this study (2.50 mg/mL), and therefore in the expected range [32].

### 2.3. Electrospun Fiber Mat Characterization

#### 2.3.1. Surface Morphology of Electrospun Fiber Mats

**Figure 2:**
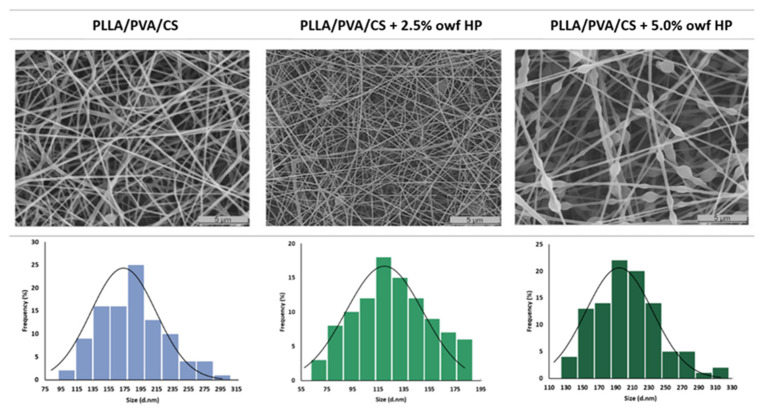
Characterization of the morphologic features of the electrospun PLLA/PVA/CS fiber mats with and without crude HP extracts. SEM images, fiber diameter, and the size distribution of the fibers.

The morphological properties of electrospun PLLA/PVA/CS fiber mats with and without crude HP extracts, produced by using uniform and homogeneous gel-like suspensions of two immiscible polymeric solutions, were analyzed using SEM. SEM micrographs and the fiber-diameter frequency distribution of the samples are shown in Figure 2. The electrospun PLLA/PVA/CS fiber mats produced from gel-like suspensions displayed fibers uniformly distributed and randomly oriented with a mean diameter of 173.57 ± 41.00 nm. Nevertheless, when 2.5% owf crude HP extract was loaded, the surface morphology exhibited thinner fibers with a mean diameter of 119.96 ± 29.90 nm. Therefore, the addition of HP extract reduced the viscosity of the electrospun solution, and consequently, the average fiber diameters decreased. However, spindles or bead-on-a-string fiber morphologies with a mean of 194.61 ± 40.30 nm were formed when the ratio of crude HP extract increased further to 5.0% owf, due to the difficulty in obtaining stable electrospun jets.

Similar results were found by Zarghami et al., who produced PCL/olive oil nanofibers with three different concentrations of olive oil (1.0%, 2.0%, and 3.0%) [23]. They found homogeneous and uniformly dispersed nanofibers when 1.0% and 2.0% olive oil were incorporated, while PCL loaded with 3.0% olive oil had difficulties producing uniform fibers. Consequently, beads and droplets alongside were observed.

Furthermore, Zarghami et al. also revealed that thinner and homogeneous nanofibers with a minimum diameter are preferred to obtain a maximum surface area and porosity structure [23]. In this context, the production of electrospun PLLA/PVA/CS fiber mats by incorporating two different weight percentages of crude HP extract demonstrated that 2.5% owf of HP is required to improve the healing process.

#### 2.3.2. Attenuated Total Reflectance-Fourier Transform Infrared Spectroscopy (ATR-FTIR) Analysis

**Figure 3:**
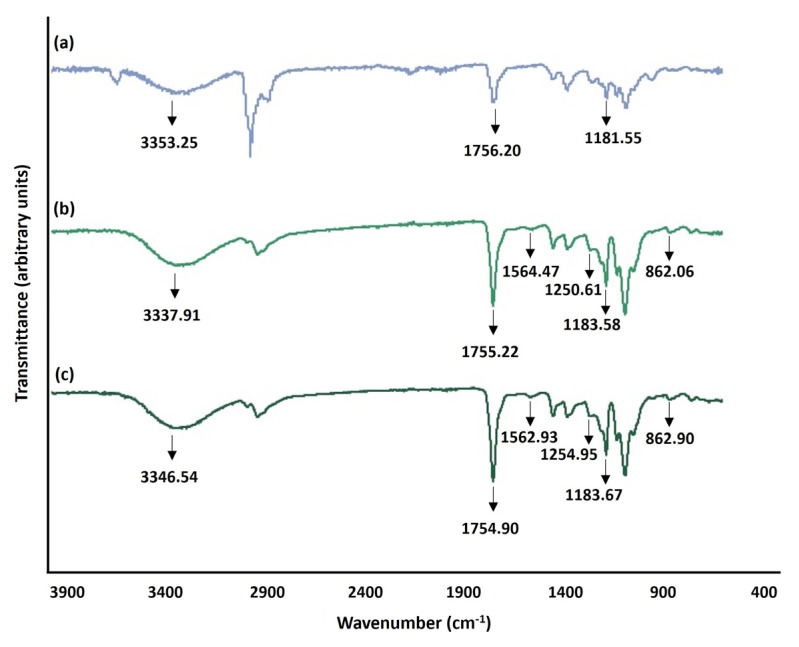
FT-IR spectra of the raw electrospun PLLA/PVA/CS fiber mats (**a**) and electrospun PLLA/PVA/CS nanofiber mats containing 2.5% and 5.0% owf crude HP extract, (**b**) and (**c**), respectively.

ATR-FTIR spectra of the electrospun PLLA/PVA/CS fiber mats produced from gel-like suspensions with and without crude HP extracts are presented in Figure 3. All the acquired spectra exhibit the characteristic bands of both CS and PVA, as well as PLLA. A broad band is observed in ATR-FTIR spectra around 3000–3500 cm^−1^ due to O-H and N-H stretching vibrations of both CS and PVA backbone [33]. On the other hand, the C=O stretching of PLLA is observed as a strong band around 1755.00 cm^−1^, while the C-O-C stretching vibration appears as a band near 1183.00 cm^−1^ [34]. Moreover, the spectra of electrospun PLLA/PVA/CS fiber mats loaded with crude HP extracts present bands at 880 cm^−1^, between 1500 cm^−1^ and 1600 cm^−1^, as well as near 1200 cm^−1^, representing the aromatic rings of HP extract, the C=C stretching vibrations, and C-O phenolic groups, respectively [30]. Furthermore, the FT-IR spectra of the electrospun PLLA/PVA/CS fiber mats suggest that the intensity of bands increases when the crude plant extract was added. Therefore, the higher intensity of the broad peak at 3400 cm^−1^ is consistent with the HP incorporated, and consequently proves the successful blending of the crude HP extracts with electrospun PLLA/PVA/CS fiber mats.

#### 2.3.3. Porosity Measurement

All the electrospun PLLA/PVA/CS fiber mats with and without crude HP extracts that were produced via emulsion electrospinning from the homogeneous gel-like suspensions of droplets of PVA/CS blend solution in a continuous PLLA aqueous solution showed a highly porous structure. The total porosity of the raw electrospun PLLA/PVA/CS fiber mats produced from gel-like suspensions was 84.52 ± 6.88%. In comparison, electrospun PLLA/PVA/CS fiber mats containing 2.5% and 5.0% owf HP presented a total porosity of 93.30 ± 1.24% and 80.03 ± 2.08%, respectively.

The slightly higher porosity value measured for the electrospun PLLA/PVA/CS fiber mats containing 2.5% HP owf can be related to the lower fiber diameters, which promote the available pore spacing between fibers. On the other hand, the difficulty in producing uniform and thinner fibers when 5.0% owf HP was loaded resulted in a lower porosity value.

Recently, Yousefi et al. produced electrospun fiber mats loaded with multiple henna extracts (0–2 wt%) [35]. Their results revealed that the incorporation of 2 wt% henna led to the production of uniform fibers with thinner diameters. Furthermore, several researchers have recently highlighted that highly porous structures with porosities above 90% are the most suitable for providing the required space for cell adhesion, spreading, and migration, which promotes the production of the new ECM [36].

In this context, 2.5% owf crude HP extract proved to be the most effective amount of crude plant extract used to display proper oxygen permeability, as well as providing nutrient and fluid exchange through the skin surface, promoting the wound-healing process.

#### 2.3.4. Water Contact-Angle Determination

WCA on the electrospun PLLA/PVA/CS fiber mats produced from gel-like suspensions with and without crude HP extracts were measured to assess the surface wettability and hydrophilicity of these materials. Based on what was previously described in the literature in relation to polymer surfaces, WCA values ranging 40–70° correspond to a moderate wettability surface, which is optimal for cell adhesion, while WCA values >90° or WCA values <20° report that the surface is hydrophobic or highly hydrophilic, respectively [37,38].

In the present study, the raw electrospun PLLA/PVA/CS fiber mats, obtained from a uniform and homogeneous gel-like suspensions of droplets of PVA/CS dispersed within a continuous PLLA solution, revealed a hydrophobic character (WCA of 94.94 ± 3.45°), while the electrospun PLLA/PVA/CS fiber mats loaded with 2.5 and 5.0% owf crude HP extracts presented WCA values of 52.22 ± 8.17° and 48.10 ± 7.01°, respectively. Such values demonstrated a more hydrophilic character when crude HP extracts were incorporated, due to their hydrophilic functional groups. Moreover, these WCA values showed a moderate wettability that is essential to improve the initial adhesion and migration of cells, as well as their proliferation to the wound site. Furthermore, moderate hydrophilic materials are suitable to assure the maintenance of an optimally moist wound environment to prevent wound dehydration [39,40].

Similarly, Jin et al. showed that the highly hydrophobic electrospun PCL fibers became more hydrophilic when plant extracts (*Indigofera aspalathoides*, *Azadirachta indica*, *Memecylon edule*, and *Myristica andamanica*) were incorporated [40]. The study also revealed that moderate hydrophilic surfaces displayed a better affinity toward cells.

#### 2.3.5. Water Vapor Transmission Rate (WVTR)

WVTR was measured to evaluate the ability of the electrospun fiber mats produced from gel-like suspensions to provide an optimal moist wound environment, which plays a vital role in the wound-healing process by preventing dehydration and fluid accumulation while enhancing the re-epithelization process [41,42].

Depending on the type of wound and stage of healing, the WVTR from skin can change in a broad range. Typically, an WVTR for healthy skin is reported as 204 g m^−2^ day^−1^ while that for injured skin can range from 279 g m^−2^ day^−1^ to 5138 g m^−2^ day^−1^ [41,42,43].

The electrospun PLLA/PVA/CS fiber mat produced through an emulsion electrospinning technique using uniform and homogeneous gel-like suspensions resulted in a WVTR of 1890.71 ± 104.50 g m^−2^ day^−1^, while electrospun PLLA/PVA/CS fiber mats loaded with 2.5% and 5.0% owf crude HP extracts reported WVTRs of 2230.58 ± 39.49 and 1729.50 ± 38.47 g m^−2^ day^−1^, respectively. Hence, the electrospun PLLA/PVA/CS fiber mats loaded with 2.5% owf HP revealed a higher WVTR than the ones achieved by using a higher crude HP extract ratio, mainly due to the high porosity and hydrophilic character.

Chitrattha et al. revealed that the hydrophilic character of PLA was improved when PEG was incorporated, consequently enhancing their WVTR values [44]. On the other hand, Alippilakkotte et al. produced PLA/silver nanofibers using electrospinning [45]. The silver-extract nanoparticles were synthesized from *Momordica charantia* fruit extract and loaded with three different concentrations (1.0 wt% (PLA-1Ag), 2.0 wt% (PLA-2Ag) and 3.0 wt% (PLA-3Ag)). Their results demonstrated that the WVTR rate of the PLA-2Ag sample was significantly lower than the PLA-1Ag and PLA-3Ag samples. The acquired data were explained by the lower porosity for this sample and are in accordance with the WVTR of the electrospun PLLA/PVA/CS fiber mats containing 5.0% owf crude HP extract. Therefore, the WVTR values obtained in the present study suggest that the electrospun PLLA/PVA/CS fiber mat loaded with 2.5% owf crude HP extract from gel-like suspensions can provide the most suitable moist environment.

#### 2.3.6. Swelling Determination

**Figure 4:**
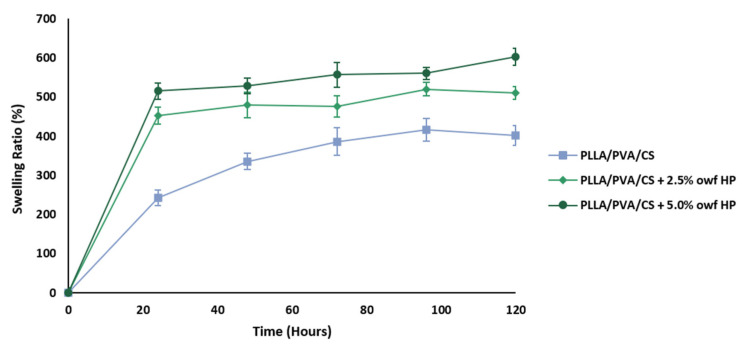
Characterization of the effect of crude HP extracts on the degree of swelling (%) in PBS (pH = 5.5) for 120 h at 37 °C.

The swelling ability of the electrospun PLLA/PVA/CS fiber mats produced from gel-like suspensions and loaded with crude HP extracts was studied in a PBS solution and over time periods of 0, 24, 48, 72, 96, and 120 h, shown in Figure 4. The curves showed a rapid swelling rate during the first 24 h, and then it leveled off as time passed. The electrospun PLLA/PVA/CS fiber mat fabricated using a homogeneous gel-like suspension via emulsion electrospinning was able to swell up to 401.19 ± 25.14%, while electrospun PLLA/PVA/CS fiber mats containing 2.5% and 5.0% owf crude HP extracts exhibited swelling rates of 510.53 ± 15.71% and 603.19 ± 21.34%, respectively. The results demonstrated that the highest swelling rate was obtained when the crude HP extract concentration increase to 5.0% owf. Such behavior can be explained by the hydrophilic character of crude HP extract, due to the presence of hydrophilic functional groups on its structure that can be easily hydrated, such as hydroxyl, amino, and carboxyl groups. Therefore, in these conditions, the fluids quickly enter into the pores of the electrospun fiber mats and gradually diffuse into the fibers while creating and maintaining a moist wound environment to facilitate the wound-healing process [46]. Moreover, the high swelling can prevent the dressing from sticking to the wound surface and improve the transport of oxygen to the wound surface, resulting in rapid, and better healing and recovery of the wound [35,47].

Sadri et al. proved that the swelling properties also enhanced with the amount of green tea leaf extract loaded [47]. Thus, polymeric nanofiber composite holds more moisture in its structure when a higher concentration of green tea is used.

### 2.4. In Vitro Release Study of HP-Loaded Electrospun PLLA/PVA/CS Fiber Mats

**Figure 5:**
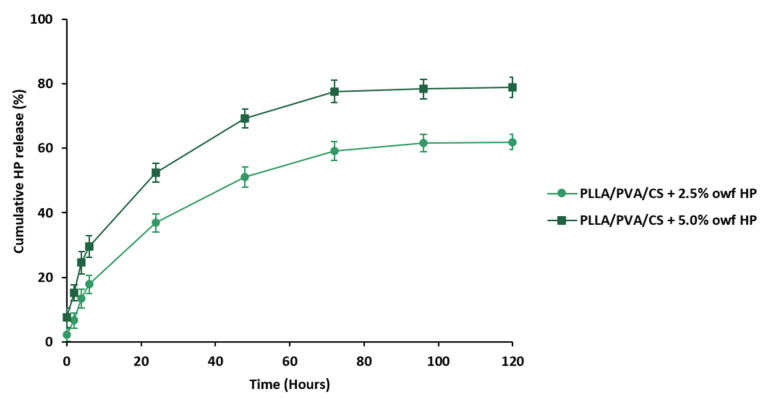
In vitro release study of crude HP extract loaded into electrospun PLLA/PVA/CS fiber mats in a PBS buffer solution (pH = 5.5) for 120 h at 37 °C.

The release profiles of crude HP extract from electrospun PLLA/PVA/CS fiber mats prepared using gel-like suspensions with different HP concentrations (2.5% and 5.0% owf) were reported by the total immersion method using PBS at pH = 5.5 as release medium, Figure 5. In vitro release profiles exhibited a sustained release of the crude HP extracts during 72 h, which is appropriate for improving the healing process.

Evidently, electrospun PLLA/PVA/CS fiber mats containing 5.0% owf HP exhibited a faster release rate (78.99 ± 3.14%) than for 2.5% owf of HP (61.89 ± 2.36%). These results suggest that the highest amount of crude plant extract loading displays a faster diffusion, and consequently, the release rate increases while enhancing the degree of swelling of the electrospun PLLA/PVA/CS fiber mats fabricated by an emulsion electrospinning technique using uniform and homogeneous gel-like suspensions.

The data obtained are in agreement with those previously described by Panichpakdee et al., who described the production of the ultra-fine cellulose acetate (CA) fiber mats loaded with emodin, an active herbal substance for use in wound healing extracted from the plant *Polygonum cuspidatum* p.e, at various amounts (0.01 wt%, 0.05 wt%, and 0.10 wt%) [15]. In both cases, the electrospun fibers containing the highest concentration of loaded extract exhibited the maximum release rate, as well as a faster release process.

### 2.5. Antibacterial Activity Assessment

The antibacterial efficiency of the electrospun PLLA/PVA/CS fiber mats produced from gel-like suspensions and loaded with crude HP extracts was successfully evaluated only against *S. aureus* (gram-positive bacterium), one of the most common bacteria isolates from chronic wounds [11].

**Figure 6:**
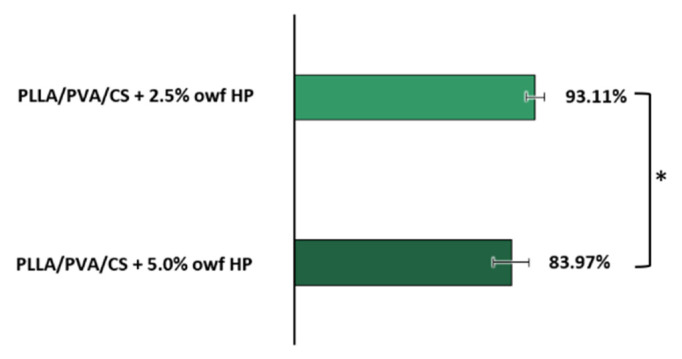
Antibacterial efficiency of crude HP extract-loaded electrospun PLLA/PVA/CS fiber mats against *S. aureus*, expressed in percentage of microbial inhibition (%R). These values were obtained by comparison with the results of the raw electrospun PLLA/PVA/CS fiber mats. (Data are presented as the mean ± SD, * *p* < 0.05).

The quantitative assay showed that after 24 h of contact with crude HP extract-loaded electrospun PLLA/PVA/CS fiber mats, there was a percentage of microbial inhibition against *S. aureus* of 93.11 ± 3.53% and 83.97 ± 6.95% when 2.5% and 5.0% owf HP were incorporated, respectively, as shown in Figure 6. In this way, the electrospun PLLA/PVA/CS fiber mats containing 2.5% and 5.0% owf HP extract presented a lower number of viable bacterial cells (1.33 × 10^5^ CFU/mL and 3.10 × 10^5^ CFU/mL, respectively) in comparison with the electrospun PLLA/PVA/CS fiber mats (1.93 × 10^6^ CFU/mL), displaying a refined antibacterial effect.

This evaluation demonstrated that 2.5% owf HP exhibits a higher ability to inhibit the bacterial growth, which translates its stability and effectiveness. Therefore, it can be concluded that the distribution of the HP in the nanofibers as well as their morphology, namely the defects and beaded structures, significantly influenced the antibacterial potential of the PLLA/PVA/CS fiber mats when 5.0% owf HP was incorporated.

Moreover, as expected, the antibacterial properties of the electrospun PLLA/PVA/CS fiber mats produced via emulsion electrospinning from homogeneous gel-like suspensions were conferred by CS’s inherent antimicrobial action and further strengthened with the adding of the crude HP extracts.

These results are in accordance with the data previously reported by Bai et al., who found that the extra loading of tea tree oil (TTO) in the PCL/Chitosan dressings increased the antimicrobial activity [48].

### 2.6. In Vitro Cytotoxicity Evaluation

**Figure 7:**
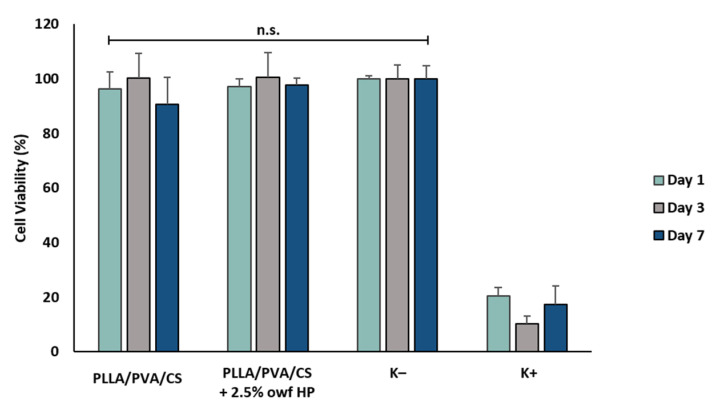
Evaluation of the in vitro cytotoxic profile of the produced electrospun PLLA/PVA/CS fiber mats with and without HP extract. Analysis of the NHDF cell viability via MTT assay after 1, 3, and 7 days.

The suitability of the produced electrospun fiber mats, through the creation of an emulsion within a single gel-like polymeric solution, for being applied as wound-dressing materials was assessed from direct cytotoxicity assay using NHDF cells. The raw electrospun PLLA/PVA/CS fiber mats and electrospun PLLA/PVA/CS fiber mats containing 2.5% owf HP, which resulted in better wound-dressing properties, were investigated. The results revealed that the presence of the crude HP extract in the fiber mats did not produce any cytotoxic effect on the viability of the NHDF cells, over 7 days, as shown in Figure 7. In both cases, the cell viability was higher than 70% (non-cytotoxic effect), confirming that the incorporation of the crude HP extract in the electrospun PLLA/PVA/CS nanofibers produced from gel-like suspensions did not affect their biocompatibility.

## 3. Conclusions

This research work describes the development of new non-woven electrospun wound-dressing materials for the treatment and avoidance of skin wound infections. These materials, composed of PLLA, PVA, and CS, were produced from W/O emulsions via emulsion electrospinning using Nanospider technology and effectively loaded with two different concentrations of crude HP extract (2.5 and 5.0% owf). The electrospun PLLA/PVA/CS fiber mats that were produced via emulsion electrospinning using uniform and homogeneous gel-like suspensions of immiscible polymers proved to be suitable to mimic the properties of native skin structure, mainly when 2.5% owf crude HP extract was incorporated. This was proposed by the fact that thinner fibers were produced with diameters within the preferred size range to the development of a new ECM. Moreover, electrospun PLLA/PVA/CS fiber mats produced from gel-like suspensions and loaded with 2.5% owf crude HP extract displayed a most proper porosity, wettability, and degree of swelling, which enable the establishment of a moist wound environment, improving the capability of absorbing and retaining the remaining wound exudate. Furthermore, this dressing material exhibited an enhanced antibacterial activity against *S. aureus* (93.11% ± 3.53%), without significantly affecting the viability of NHDF cells.

Therefore, the obtained results showed that electrospun PLLA/PVA/CS fiber mats containing 2.5% crude HP are a promising approach for the development of antibacterial wound dressings. Moreover, in a near future, a droplet-size tracking study and a rheological report of the emulsions produced from gel-like suspensions of two naturally immiscible polymer solutions should be further accomplished to aid the formation of the emulsions and ensure their stability and homogeneity. In addition, the membranes’ porosity may be determined using different displacement liquids to ensure their solubility, and additional in vitro and in vivo studies may be performed to predict the efficacy and safety of the produced electrospun wound-dressing materials.

## 4. Materials and Methods

### 4.1. Materials

The aerial parts of *Hypericum perforatum* L. (HP) were bought from a Portuguese botanic shop. Poly(L-lactic acid) (PLLA) with an average molecular weight of 217,000–225,000 g/mol, Purasorb^®^ (PL18) was provided by Corbion Purac. Poly (vinyl alcohol) (PVA) (MW 115,000 g/mol) was purchased from VWR Chemicals, and Chitosan (CS) (MW 50,000–190,000 g/mol, degree of deacetylation 75–85%) was obtained from Sigma-Aldrich. Chloroform (analytical grade), dimethylformamide (DMF) (analytical grade), glacial acetic acid, and ethanol absolute were purchased from Fisher Chemical. Nutrient agar (NA), nutrient broth (NB), and agar for microbiology were obtained from Fluka. Sodium chloride (NaCl), Mueller–Hinton broth (MHB), tween 80, dimethyl sulfoxide (DMSO) anhydrous ≥99.9%, trypsin, and 3-(4,5-Dimethyl-2-thiazolyl)-2,5-diphenyl-2H-tetrazolium bromide (MTT) were provided by Sigma Aldrich. Normal human dermal fibroblast (NHDF) cells were acquired from ATCC—American Type Culture Collection. Phosphate-buffered saline (PBS) was purchased from Alfa Aesar. All solvents were used as received without further purification.

### 4.2. Crude Ethanol HP Extract

#### 4.2.1. Preparation of Crude HP Extract

Ethanol extracts from the aerial parts of the HP species were obtained via direct maceration of dried and powdered plant material (2.50 g) in 80% ethanol for 24 h at room temperature. Extracts were filtered, and the solvent was evaporated using Rotavapor (Buchi Rotavapor RE 111, Flawil, Switzerland) to provide dried extracts. The extract yield percentage based on the starting material was 20.44%. Finally, the dried HP extract was used for all experiments, as described below.

#### 4.2.2. Quantification of Total Hypericin (Hyp) Content in Crude Ethanol HP Extract

The total content of Hyp, one of the main bioactive agents present in the crude HP extract, was analyzed using the spectrophotometric method. For this purpose, standard solutions of dried crude HP extract with concentrations from 6000 to 50 ppm were prepared. Then, the Hyp content (% Hyp (*w*/*w*)) was determined by the optical absorbance of each HP solution at 587 nm, using phosphate buffer saline (PBS) pH = 5.5 as control. These measurements were performed as described by Pourhojat et al. and calculated using the following Equation (1) [30]:

(1) $$\mathrm{Hyp}\;\left(\%\right)=\frac{A}{780}\frac{100}{m}$$

where A is the measured absorbance, m the weight of the HP extract after drying of 25 mL of crude extract, and 780 the specific absorbance of Hyp at 587 nm.

### 4.3. Determination of Minimum Inhibitory Concentration (MIC) of Crude Ethanol HP Extract

The minimal inhibitory concentration (MIC) of crude ethanol HP extract against *Staphylococcus aureus* (ATTC 6538) (gram-positive bacterium) (*S. aureus*), was measured by broth microdilution assay according to NCLS M07-A6 guidelines. Briefly, crude ethanol HP extract stock solution was prepared to a final concentration of 20 mg/mL and then serially diluted with sterile Mueller–Hinton Broth (MHB) ranging from 0.1 to 10 mg/mL.

Afterward, an overnight liquid culture of *S. aureus* was adjusted to 0.5 McFarland turbidity standards using sterile water and the bacterial work suspension prepared from 500 µL of the 0.5 McFarland suspension and 4500 µL of MHB. Then, 50 µL of bacterial work suspension and 50 μL of the crude ethanol HP extract dilutions were added into 96 multi-well plates.

### 4.4. Preparation of Electrospinning Emulsions

CS (4% *w*/*v*) and PVA (8% *w*/*v*) solutions were initially prepared separately by dissolving CS in acetic acid (14%) at room temperature and PVA in deionized water at 90 °C. Then, the PVA/CS binary blend was obtained by the blending of PVA solution and CS gel solution with a ratio of 2:1 and stirring at room temperature during a few minutes. Additionally, different amounts of crude HP extract were added to the PVA/CS gel-like blend to obtain solutions containing 2.5 and 5.0% HP (based on the weight of PVA/CS blend (owf)) which corresponds to 3 mg/mL and 6 mg/mL of HP extract, respectively.

In addition, PLLA (10% *w*/*v*) was dissolved in chloroform and DMF (9:1 volume ratio) at room temperature using a magnetic stirrer until complete polymer dissolution.

The emulsions were prepared by the simultaneous adding of the PVA/CS binary blend gel-like solution, with and without HP extract, to the PLLA solution, followed by mixing with a high-speed homogenizer (Techmatic S2) with continuous stirring at 1000 rpm for 4 h at room temperature to ensure complete dissolution and to obtain uniform and homogenous gel-like suspensions of two naturally immiscible polymer solutions. The polymeric blends were cooled during the emulsion’s preparation using an ice bath to prevent excessive heating and to avoid the evaporation of the plant’s volatile components.

### 4.5. Electrospinning

#### Preparation of Electrospun PLLA/PVA/CS Fiber Mats Containing Crude HP Extracts

The uniform and homogenous PLLA/PVA/CS emulsions prepared using gel-like suspensions of two immiscible polymeric solutions were electrospun using Nanospider technology (Nanospider laboratory machine NS LAB 500S from Elmarco s.r.o., Czech Republic, http://www.elmarco.com, accessed on 2 February 2023). Electrospun fiber mats containing different amounts of crude HP extract were also produced. For this, 3 mg/mL and 6 mg/mL of the crude HP extracts were added to the PVA/CS gel-like blend to obtain solutions containing 2.5 and 5.0% owf of PVA/CS gel-like blend, as previously described in Section 4.4. All resulting solutions were electrospun at an applied voltage of 80.0 kV, using a working distance (distance from the electrode to collector) of 10 cm and an electrode rotation rate of 70 Hz (electrode spin = 11.2 r/min). Each solution was electrospun for 1 h on polypropylene non-woven fabric (collector) at 25 °C and relative humidity up to 35%. After spinning, the produced electrospun PLLA/PVA/CS fiber mats with and without crude HP extracts were dried and stored in a vacuum desiccator until constant weight and then used for characterization and bioactive experiments.

### 4.6. Nanofiber Characterization

#### 4.6.1. Surface Morphology of Electrospun Fiber Mats

The morphology of the electrospun PLLA/PVA/CS fiber mats prepared from gel-like suspensions with and without crude HP extracts was observed using a scanning electron microscope (SEM; Hitachi S2700, Düsseldorf, Germany) at a high voltage of 20 kV. The fiber diameters were analyzed with the help of the Image J software (National Institutes of Health, Bethesda, Montgomery, MD, USA), while both average diameter and diameter distribution were evaluated using GraphPad Prism 6 software (Prism Software, Irvine, CA, USA).

#### 4.6.2. Fourier Transform Infrared Spectroscopy (FT-IR)

The differences in chemical structure between the electrospun PLLA/PVA/CS fiber mats produced from gel-like suspensions with and without crude HP extracts were analyzed using Thermo-Nicolet is10 FT-IR spectrophotometer over the range 400–4000 cm^−1^. FT-IR spectra were collected from 64 scans at 4 cm^−1^ resolution.

#### 4.6.3. Porosity Measurement

The porosity values of dry raw electrospun PLLA/PVA/CS fiber mats produced from gel-like suspensions with and without crude HP extracts were determined using the liquid displacement technique described previously by Chitrattha et al. [44]. Ethanol was chosen as the displacement liquid due to its high penetrating power into the pores and because it did not cause shrinking or swelling of the samples.

Briefly, the dried samples were weighed (W_s_) and placed in a graduated cylinder with a known volume (20 mL) of displacement liquid (W_1_). Then, the cylinders containing the electrospun fiber mats immersed into the displacement liquid were placed in a water sonicator bath (Ultrasons-H, P-Selecta) for 40 min at 30 °C to force the penetration of ethanol into the pores. After that, the volume of displacement liquid in the sonicated cylinders containing the ethanol-impregnated samples was readjusted to 20 mL and reweighed (W_2_). Subsequently, the cylinders were weighed again after removing the saturated samples with ethanol (W_3_). The total porosity (ε) of the electrospun fiber mats was determined using the following Equation (2) [44]:

(2) $$\varepsilon \;\left(\%\right)=\frac{\left(W_{2}-W_{3}-W_{S}\right)}{\left(W_{1}-W_{3}\right)}\times 100$$

#### 4.6.4. Water Contact-Angle Determination

The static contact angles for water (WCA) on the electrospun PLLA/PVA/CS fiber mats produced from gel-like suspensions with and without crude HP extracts were measured using Data Physics Contact Angle System OCAH-200 apparatus to characterize the surface wettability. For each sample, the contact angle was determined from sessile drop method, i.e., deionized water droplets were deposited on the surface of the dried electrospun fiber mats at room temperature. The contact angles were reported as the average of three independent measurements.

#### 4.6.5. Water Vapor Transmission Rate (WVTR)

Water vapor diffusion rates were measured following the ASTM E96/E96M-15 standard method for all the samples, i.e., raw electrospun PLLA/PVA/CS fiber mats prepared from gel-like suspensions, and electrospun PLLA/PVA/CS fiber mats loaded with crude HP extracts. Briefly, each sample was attached to the opening of a glass test tube (1.20 cm in diameter) with 10 mL of deionized water using parafilm tape to prevent any moisture loss. Subsequently, the samples–glass tubes assembly was incubated at 37 °C. The water evaporation from each sample was determined by weight loss at predetermined intervals through Equation (3):

(3) $$\mathrm{Water}\;\mathrm{Vapor}\;\mathrm{Transmission}\;\mathrm{Rate}\;\left(\mathrm{WVTR}\right)=\frac{W_{\mathrm{loss}}}{A}$$

where W_loss_ is the daily weight loss of water, and A is the area of the glass tube opening. All the measurements were performed in triplicate.

#### 4.6.6. Swelling Determination

The swelling behavior of the electrospun PLLA/PVA/CS fiber mats produced from gel-like suspensions with and without crude HP extracts was evaluated using the water retention test. Briefly, pre-weighted dried samples (W_dry_) were immersed in phosphate-buffered saline (PBS, pH = 5.5) during 120 h at 37 °C, which plays a crucial role in the maintenance of healthy skin microbiome and has beneficial wound-healing effects. At specific periods, the weights of the swollen samples (W_wet_) were determined after removing the PBS solution excess present at the surface of each sample carefully with a filter paper. All measurements were conducted in triplicate, and the swelling ratio calculated using Equation (4):

(4) $$\mathrm{welling}\;\mathrm{Ratio}\;\left(\%\right)=\frac{\left(W_{\mathrm{wet}}-W_{\mathrm{dry}}\right)}{W_{\mathrm{dry}}}\times 100$$

### 4.7. In Vitro Release Study of HP-Loaded Electrospun PLLA/PVA/CS Fiber Mats

In vitro releasing of crude HP extracts from electrospun PLLA/PVA/CS fiber mats produced from gel-like suspensions containing different amounts of HP was carried out and analyzed using UV-Vis spectrometry. To accomplish this, standard HP solutions were prepared with different concentrations to draw a calibration curve at 587 nm [30]. Afterward, 2 cm × 2 cm samples were immersed in 10 mL of PBS at pH = 5.5 and then incubated in an incubator shaker at 37 °C under constant rotation at a speed of 100 rpm to measure the HP amount released into the PBS release medium. After specific time intervals, 3 mL of release medium was recovered and, at the same time, replenished with an equal volume of the fresh PBS. The HP concentration that remained in the release medium at each time point was quantified at 587 nm using a UV-Vis spectrophotometer. All measurements were carried out in triplicate.

### 4.8. Antibacterial Activity Assessment

The antibacterial effect of crude HP extracts loaded into electrospun PLLA/PVA/CS fiber mats produced from gel-like suspensions was tested through ASTM E2180-07. Briefly, a bacterial suspension (*S. aureus* (ATTC 6538), 1–5 × 10^8^ CFU/mL) was prepared from overnight liquid culture, added in an agar slurry (0.85 (*w*/*v*) NaCl and 0.30 (*w*/*v*) agar-agar in deionized water) and then spread over the 3 cm × 3 cm square samples. The samples were assessed immediately after inoculum application (T_0h_) and after 18–24 h in contact with the inoculum agar slurry at 37 °C for 18–24 h (T_24h_). To accomplish this, 0.85 (*w*/*v*) NaCl was used to perform serial dilutions and then plated in the NA medium and incubated for 18–24 h at 37 °C.

The antimicrobial activity was calculated to determine bacterial growth inhibition (%Inhibition) using the following Equation (5):

(5) $$\mathrm{Bacterial}\;\mathrm{growth}\;\mathrm{inhibition}\;\left(\%\right)=\frac{C-S}{C}\times 100$$

where C is the average value of colony forming units (CFU) on the control sample (electrospun PCL/PVA/CS fiber mat), and S is the average value of CFU on the samples containing different amounts of crude HP extract (electrospun HP-loaded PLLA/PVA/CS fiber mats) [49].

### 4.9. In Vitro Cytotoxicity Evaluation

Changes in cell viability produced by the electrospun fiber mats prepared from gel-like suspensions were evaluated by direct cytotoxicity assay using 3-(4,5-dimethylthiazol-2-yl)-2,5-diphenyltetrazolium bromide (MTT) colorimetric method following ISO 10993–5 (Biological evaluation of medical devices-Part 5: Tests for in vitro cytotoxicity). Briefly, the samples were placed in 24-well plates occupying <10% of its area and then sterilized using UV irradiation (254 nm, ~7 mW cm^−2^) for 1 h. After that, normal human dermal fibroblasts (NHDF) cells were seeded onto the samples at a cell density of 1 × 10^4^ cells/well and then incubated for 1, 3, and 7 days at 37 °C, in an incubator under a 5% CO_2_ humidified atmosphere. After the incubation period, the cellular metabolic activities were evaluated through the cleavage of the MTT tetrazolium ring and formation of the formazan blue crystals. For this purpose, the medium of each well was removed and then a mixture of fresh culture medium containing the MTT solution added to each well. After 4 h of incubation, the content of each well was replaced by DMSO to dissolve the formazan crystals. After shaking, the optical densities (OD) of each sample (n = 5) were measured at 570 nm using a microplate reader (Biorad xMark microplate spectrophotometer, Algés, Portugal). Cells in the culture medium only and cells cultured in EtOH (96%) were included as negative (K^−^) and positive (K^+^) controls, respectively.

### 4.10. Statistical Analysis

The statistical analysis of the results obtained was carried out using analysis of variance (ANOVA) followed by multiple comparison tests using Tukey’s test at the 95% confidence level. Statistical analyses were performed in GraphPad Prism 6 software (Prism Software, USA).

## Figures and Tables

**Table 1:** Determination of Hypericin (Hyp) content in crude ethanol extract of HP.

Samples	Dried Extract (ppm)	Dried Extract (g)	Absorbance in λ_587_	Hyp (%)
1	6000	0.30	0.86	0.37
2	4000	0.20	0.64	0.41
3	3000	0.15	0.39	0.33
4	1000	0.05	0.07	0.19
5	500	0.025	0.04	0.21
6	200	0.01	0.02	0.27
7	100	0.005	0.01	0.26
8	50	0.0025	0.01	0.33
Average = 0.30 ± 0.07				

## Data Availability

Not applicable.
